# The Temple University Hospital EEG Data Corpus

**DOI:** 10.3389/fnins.2016.00196

**Published:** 2016-05-13

**Authors:** Iyad Obeid, Joseph Picone

**Affiliations:** Electrical and Computer Engineering, Temple UniversityPhiladelphia, PA, USA

**Keywords:** EEG, database, machine learning, clinical trials as topic, big data

## Introduction

The electroencephalogram (EEG) is an excellent tool for probing neural function, both in clinical and research environments, due to its low cost, non-invasive nature, and pervasiveness. In the clinic, the EEG is the standard test for diagnosing and characterizing epilepsy and stroke, as well as a host of other trauma and pathology related conditions (Tatum et al., 2007; Yamada and Meng, 2009). In research laboratories, EEG is used to study neural responses to external stimuli, motor planning and execution, and brain-computer interfaces (Lebedev and Nicolelis, 2006; Wang et al., 2013). While human interpretation is still the gold standard for EEG analysis in the clinic, a host of software tools exist to facilitate the process or to make predictive analyses such as seizure prediction.

Recently, a confluence of events has underscored the need for robust EEG tools. First, there has been a renewed push via the White House BRAIN initiative to understand neural function and disease (Weiss, 2013). Secondly, there is an increased awareness on brain injury owing to both the influx of injured warfighters and numerous high-profile athletes found to have chronic brain damage (McKee et al., 2009; Stern et al., 2011). And thirdly, a wave of consumer grade scalp sensors has entered the market, allowing end users to monitor sleep, arousal, and mood (Liao et al., 2012).

In all these applications, there is a need for robust signal processing tools to analyze the EEG data. Historically, EEG signal processing tools have been devised using either ad hoc heuristic methods, or by training pattern recognition engines on small data sets (Gotman, 1982). These methods have yielded limited results, owing mostly to the fact that brain signals (and EEG in particular) are characterized by great variability, which can only be properly interpreted by building statistical models using massive amounts of data (Alotaiby et al., 2014; Ramgopal et al., 2014). Unfortunately, despite EEG being perhaps the most pervasive modality for acquiring brain signals, there is a severe lack of data in the public domain. For example, the “EEG Motor Movement/Imagery Dataset” (http://www.physionet.org/pn4/eegmmidb/) contains ~1500 recordings of 1 or 2 min duration apiece from 109 subjects (Goldberger et al., 2000; Schalk et al., 2004). The CHB-MIT database contains data from 22 subjects, mostly pediatric (Shoeb, 2009). A database from Karunya University contains 175 16-channel EEGs of duration 10 s (Selvaraj et al., 2014). One of the most extensive databases for supporting epilepsy research is the European Epilepsy Database (http://epilepsy-database.eu/), which contains 250 datasets from 30 unique patients, but sells for €3000. Other databases, such as ieee.org, contain a wealth of data from more invasive modalities such as electrocorticogram, but little or no EEG.

This lack of publically available data is ironic considering that hundreds of thousands of EEGs are administered annually in clinical settings around the world. Relatively little of this data is publicly available to the research community in a form that is useful to machine learning research. Massive amounts of EEG data would allow the use of state-of-the-art machine learning algorithms to discover new diagnostics and validate clinical practice. Furthermore, it is desirable that such data be collected in clinical settings, as opposed to tightly controlled research environments, since “clinical-grade” data is inherently more variable with respect to parameters such as electrode location, clinical environment, equipment, and noise. Capturing this variability is critical to the development of robust, high performance technology that has real-world impact.

In this work, we describe a new corpus, the TUH-EEG Corpus, which is an ongoing data collection effort that has recently released 14 years of clinical EEG data collected at Temple University Hospital. The records have been curated, organized, and paired with textual clinician reports that describe the patients and scans. The corpus is publicly available from the Neural Engineering Data Consortium (www.nedcdata.org) (Picone and Obeid, 2016).

## Methods

Clinical EEG data were collected from archival records at Temple University Hospital (TUH). All work was performed in accordance with the Declaration of Helsinki and with the full approval of the Temple University IRB. All personnel in contact with privileged patient information were fully trained on patient privacy and were certified by the Temple IRB.

Archival EEG signal data were recovered from CD-ROMs. Files were converted from their native proprietary file format (Nicolet&s NicVue) to an open format EDF standard. Data was then rigorously de-identified to conform to the HIPAA Privacy Rule by eliminating 18 potential identifiers including patient names and dates of birth. Patient medical record numbers were replaced with randomized database identifiers, with a key to that mapping being saved to a secure off-line location. Importantly, our process captured instances in which the same patient received multiple EEGs over time and assigned database IDs accordingly. Data de-identification was performed by combining automated custom-designed software tools with manual editing and proofreading. All storage and manipulation of source files was conducted on dedicated non-network connected computers that were physically located within the TUH Department of Neurology.

We also manually paired each retrieved EEG with its corresponding clinician report. These reports are generated by the neurologist after analyzing the EEG scan and are the official hospital summary of the clinical impression. These reports are comprised of unstructured text that describes the patient, relevant history, medications, and clinical impression. Reports were mined from the hospital&s central electronic medical records archives and typically consisted of image scans of printed reports. Various levels of image processing were employed to improve the image quality before applying optical character recognition (OCR) to convert the images into text. A combination of software and manual editing was used to scrub protected health information (PHI) from the reports and to correct errors in OCR transcription. Only sessions with both an EEG and a corresponding clinician report were included in the final corpus.

**Figure 1:**
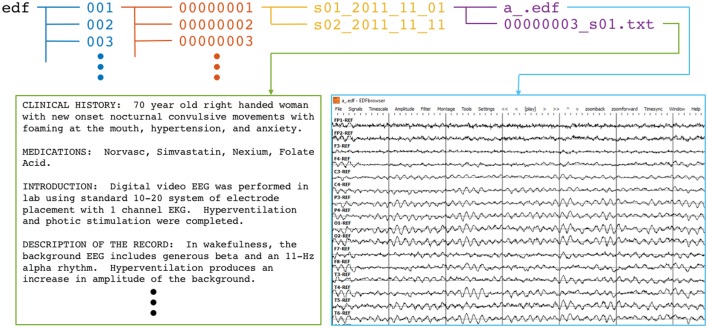
**Directory and file structure of the TUH-EEG database**. Data is organized by patient (orange) and then by session (yellow). Each session contains one or more signal (edf) and physician report (txt) files. To accommodate file system management issues, patients are grouped into sets of about 100 (blue).

The corpus was defined with a hierarchical Unix-style filetree structure. The top folder, edf, contains 109 numbered folders, each of which contain numbered folders for up to 100 patients. Each of these patient folders contains sub-folders that correspond to individual recording sessions. Those folder names reflect the session number and date of recording. Finally, each session folder includes one or more EEG (.edf) data files as well as the clinician report in .txt format. Figure 1 summarizes the corpus file structure and gives examples of text and signal data.

## Results

**Figure 2:**
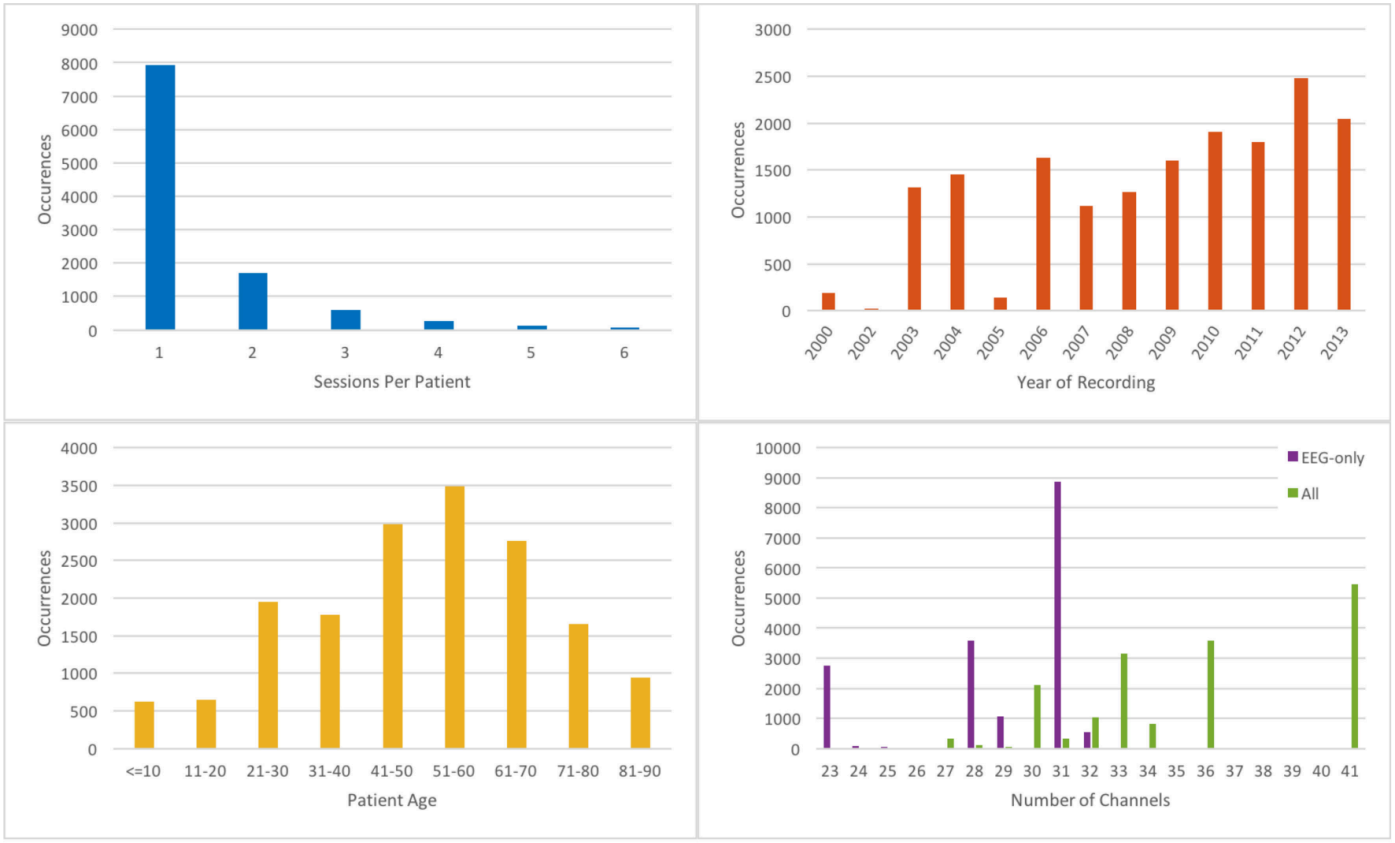
**Metrics describing the TUH-EEG corpus**. [**Top left**] histogram showing number of sessions per patient; [**top right**] histogram showing number of sessions recorded per calendar year; [**bottom left**] histogram of patient ages; [**bottom right**] histogram showing number of EEG-only channels (purple); and total channels (green).

The completed corpus comprises 16,986 sessions from 10,874 unique subjects. Each of these sessions contains at least one EDF file (more in the case of long term monitoring sessions that were broken into multiple files) and one physician report. Corpus metrics are summarized in Figure 2. Subjects were 51% female and ranged in age from less than 1 year to over 90 (average 51.6, stdev 55.9; see Figure 2 bottom left). The average number of sessions per patient was 1.56, although as many as 37 EEGs were recorded for a single patient over an 8-month period (Figure 2 top left). The number of sessions per year varies from ~1000 to 2500 (with the exception of years 2000–2002, and 2005, in which limited numbers of complete reports were found in the various electronic medical record archives; see Figure 2 top right).

There was a substantial degree of variability with respect to the number of channels included in the corpus (see Figure 2 bottom right). EDF files typically contained both EEG-specific channels as well as supplementary channels such as detected bursts, EKG, EMG, and photic stimuli. The most common number of EEG-only channels per EDF file was 31, although there were cases with as few as 20. A majority of the EEG data was sampled at 250 Hz (87%) with the remaining data being sampled at 256 Hz (8.3%), 400 Hz (3.8%), and 512 Hz (1%).

An initial analysis of the physician reports reveals a wide range of medications and medical conditions. Unsurprisingly, the most common listed medications were anti-convulsants such as Keppra and Dilantin, as well as blood thinners such as Lovenox and heparin. Approximately 87% of the reports included the text string “epilep,” and about 12% included “stroke.” Only 48 total reports included the string “concus.”

The TUH-EEG corpus v0.6.0 has been released and is freely available online at www.nedcdata.org. Users must register with a valid email address. The uncompressed EDF files and reports together comprise 572 GB. For convenience, the website stores all data from each patient as individual gzip files with a median filesize of 4.1 MB; all 10,874 gzips together comprise 330GB. Users wanting to access the entire database are encouraged to physically mail a USB hard drive to the authors in order to avoid the downloading process.

## Discussion

This work presents the world&s largest publically available corpus of clinical EEG data, representing a grand total of 29.1 years (total duration summed over all EEG channels) of EEG data. In addition to its size, this corpus features a wide variation of patient ages, diagnoses, medications, channel counts, and sampling rates. Furthermore, the corpus continues to be expanded at a rate of ~2500 new sessions per year.

Biomedicine is entering a new age of data-driven discovery driven by ubiquitous computing power, inexpensive data storage, the machine learning revolution, and high speed internet connections. Access to massive quantities of properly curated data is now the critical bottleneck to advancement in many areas of biomedical research. Ironically, doctors and clinicians generate enormous quantities of data every day, but that information is almost exclusively sequestered in secure archives where it cannot be used for research by the biomedical research community. The quantity, quality, and variability of such data represent a significant unrealized potential, which is doubly unfortunate considering that the cost of generating that data has already been borne. Although, there has been some advancement with respect to publishing databases of patient metadata, curated *signal* databases are much less commonly available, especially in quantities that would be sufficient to train most contemporary machine learning engines.

In this work, we have endeavored to achieve two goals. The first is to create a corpus of clinical EEG signals and their corresponding physician reports. The second is to establish best practices for the curation and publication of clinical signal data, which is an inherently different entity than discrete metadata. The EEG corpus we present here is the first of its kind, both in terms of volume and heterogeneity, both of which are critical factors for training machine learning engines. Typically, “research-grade” data is created by tightly controlling as many external factors as possible. In contrast, “clinical-grade” data is inherently heterogeneous with respect to those same external factors. Whereas certain classes of research questions can only be answered using well-controlled data, others benefit from variability. For example, an epilepsy detection algorithm that is trained using 31 specific EEG channels may not be effective if one or more of those channels are not connected, or if the electrodes are improperly located or affixed to the scalp. Algorithms that must be sufficiently robust to function under a plurality of conditions must be trained with data that is sufficiently heterogeneous.

Our work has shown that, although clinical signal data is ubiquitous and inherently valuable to the research community, it requires substantial manipulation before it can be released as an adequately curated data corpus. This effort is non-trivial, both in terms of time and cost. Our team&s activities ranged from the mundane (e.g., manually copying archival hospital data from over 1500 CD-ROMs) to more technical challenges (e.g., developing software for detecting data entry errors in the clinical records). Physician reports had to be located through one of five different EMR portals, often manually. A battery of tests was created to validate that each record was complete, unique, error-free, and completely free of privileged patient information. A rigorous accounting system was created to track and organize the tens of thousands of files and their status.

The cost to develop the TUH EEG Corpus has been relatively low, totaling less than $100 K in direct charges. As medical record technology improves, the cost of this collection can be reduced even further. On the balance, these types of large-scale collections are a worthwhile investment, since costs are minor relative to the cost of acquiring the data or conducting research on the data. In general, the authors expect that a dedicated community-wide data facility would be best suited to curate data of the magnitude and complexity described here because there are significant on-going costs associated with such an activity.

An example of these on-going costs is annotation of the data—a critical issue for machine learning research. In most semi-supervised machine learning applications, one of the first steps is to annotate the data, a process in which important elements of the signal are marked as such. This can be performed either manually by a human domain expert, or automatically with a bootstrap-style algorithm. In addition to the EEG data itself, we are releasing a collection of annotations which may be downloaded separately if they are of interest to the user. The annotations contain the start and stop time and an event label and are specific to each channel. Six classes of events are included: (1) spike and/or sharp waves (SPSW), (2) periodic lateralized epileptiform discharges (PLED), and (3) generalized periodic epileptiform discharges (GPED). SPSW events are epileptiform transients that are typically observed in patients with epilepsy. PLED events are indicative of EEG abnormalities and often manifest themselves with repetitive spike or sharp wave discharges that can be focal or lateralized over one hemisphere. These signals display quasi-periodic behavior. GPED events are similar to PLEDs, and manifest themselves as periodic short-interval diffuse discharges, periodic long-interval diffuse discharges and suppression-burst patterns according to the interval between the discharges. Triphasic waves, which manifest themselves as diffuse and bilaterally synchronous spikes with bifrontal predominance, typically at a rate of 1–2 Hz, are also included in this class.

Three events are used to model background noise: (1) artifacts (ARTF) are recorded electrical activity that is not of cerebral origin, such as those due to the equipment, patient behavior or the environment; (2) eye movement (EYEM) are common events that can often be confused with a spike; (3) background (BCKG) is used for all other signals.

These six classes (three signal classes and three noise classes) were arrived at through several iterations of a study conducted with Temple University Hospital neurologists. Automatic labeling of these events allows a neurologist to rapidly search long-term EEG recordings for anomalous behavior. However, there are many more annotations that need to be developed for this data. For example, we are currently developing technology to automatically annotate seizures. There are many other events of interest that need annotation (e.g., sleep states). We expect to be continually enhancing the value of the TUH EEG Corpus.

## Author contributions

JP led the database creation effort and co-wrote the manuscript. IO contributed to the database creation effort, performed data metrics, and co-wrote the manuscript.

## Funding

This work has been supported by DARPA award D13AP00065, NSF awards 1305190 and 1458411, and by the Research Office and Dean of Engineering at Temple University.

### Conflict of interest statement

The authors declare that the research was conducted in the absence of any commercial or financial relationships that could be construed as a potential conflict of interest.
